# The Administration of Arsenic in Skin Diseases

**Published:** 1890-06

**Authors:** M. B. Hutchins

**Affiliations:** Atlanta, Ga.; Late House Physician New York Skin and Cancer Hospital. Lecturer on Dermatology in the Atlanta Medical College


### THE ADMINISTRATION OF ARSENIC IN SKIN DISEASES.

BY M. B. HUTCHINS, M. D.,

Late House Physician New York Skin and Cancer Hospital. Lecturer on Dermatology in the Atlanta Medical College.

Dr. L. D. Bulkley, of New York, has written a paper upon the "Use and Value of Arsenic in Skin Diseases," but I have not been able to obtain it for reference. The present article is not intended as original matter, nothing here written possessing that quality save the text, being only an attempt at compilation of the views of various authorities who have written during the past forty years.

PHYSIOLOGICAL AND PATHOLOGICAL EFFECTS ON THE SKIN.—Bartholow[a] says arsenic may, in large doses, produce itching of the eyelids, urticaria, eczema, pityriasis, psoriasis, and falling of the nails and hair. Physiological experiments have been made with arsenic, upon the frog, by Drs. Ringer, Murrell and Miss Nunn, of England,[b] demonstrating its powerful action upon the epidermic layers, resulting in complete desquamation, progressing outward. Poisoning in the human subject was followed by universal desquamation.[b] Cutaneous eruptions from arsenic, characterized by nearly every form of lesion, have been described by numerous authors, but Hans Hebra[c] says neither he nor his father, who prescribed immense doses, ever saw a case, yet he admits that it may produce a macular erythema. Morrow[d] says alopecia areata has been developed by the prolonged administration of arsenic.

Hutchinson[e] has suggested that full doses of arsenic for a long period may produce epithelial cancer. He quoted cases of five patients in support of this theory, all of which had taken the drug for chronic skin disease in which it was indicated.

SOME REMARKS UPON THE INDISCRIMINATE USE OF ARSENIC IN DISEASES OF THE SKIN.—[f]About forty years ago the elder Hebra expressed his want of confidence in the "so-called specific" remedies for skin diseases. Twenty years later he wrote that he was unable to agree with the English and French "who attributed *undefined* blood purifying and eczema-curing properties to arsenic," depending himself almost entirely upon local remedies to effect a cure. Duhring[g] says it is a remedy of very great value, most so of all internally, but the case must be selected in order to attain success, 'and to say arsenic is of use in skin diseases, viewed collectively, would be a vague and meaningless assertion of no practical value.' Crocker[b] says "arsenic is used by too many as if it were a panacea for all cutaneous woes, which is far from being the case, it being often positively injurious, and to get good results it must be used intelligently and with a definite aim as to its intended *modus operandi*." Morrow[d] says the common practice of giving arsenic indiscriminately in *eczema*, irrespective of stage or character, is positively pernicious. Hans Hebra[c] says arsenic is often prescribed internally without result.

EFFECTS IN INDIVIDUAL SKIN DISEASES—ECZEMA.—Keeping in mind the fact that the action of arsenic, internally, upon the skin, is stimulant and irritative, rendering cell proliferation more active, we must agree in the almost universal verdict of authorities that arsenic would not only not benefit, but in many instances would aggravate, an acute eczema; and this applies with equal force to all acutely inflammatory diseases of the skin. Crocker[b] thinks it scarcely ever indicated or even desirable in eczema. Stelwagon[h] says arsenic seems to exert a special action in *some cases* of eczema. Neligan[i] recommended it in "lichen agrius,"

now recognized as a papulo-vesicular eczema.[b] "Lichen circinatus" seems now acknowledged to be a form of eczema—papular. Roberts[j] says arsenic is almost a specific in this disease. Piffard[k] says it will not cure eczema, but may be used in late stages as a tonic. Duhring[g] considers it of benefit in certain persistent localized papular and abortive vesicular eczemas; and chronic infantile eczema, not accompanied by digestive troubles, may be favorably influenced by its use. He says, however, that too much must not be expected of this remedy in eczema, as many cases are in no way improved and others are aggravated by its use. Van Harlingen[l] quotes Cheron (Lond. Path. Soc., 1873) as having recommended about 1/4 gr. arseniate of sodium t.i.d. for eczema in women about the time of the menopause. H. Hebra[c] thinks the action of arsenic in chronic localized eczema unworthy of mention, but it may be given with benefit in frequently relapsing general eczema. Considers it rarely indicated in children, while Morrow[d] says it is especially well borne by them, and that Wilson considered it a specific in infantile eczema.

The majority of authors think it of value in chronic, squamous eczema, probably on the theory of what Bartholow calls its substitutive action; and it may be useful in the chronic papular. The value of opinions upon its utility in these latter forms must be influenced by the views quoted against its *use* in eczema.

PSORIASIS.—As long ago as the time of Neligan,[j] arsenic had acquired some reputation as a specific in this disease. But he believed that the effect was better if some preparation of iodine, or iodine itself, were combined, and that iodine or its preparations would cure without the use of arsenic. He prescribed the former about as universally, in skin diseases, as many practitioners yet administer arsenic. The consensus of opinion is that arsenic is almost, if not quite, a specific in psoriasis, but recommendations of it are generally qualified by the injunction not to use it until the disease has passed into the chronic stage, on the already mentioned ground of its irritant action on the skin. When indicated, the remedy must be pushed to large doses, or to toleration. There often occurs a transitory appearance of aggravation of the skin symptoms, but this is soon followed by marked evidences of improvement. H. Hebra[c] says it is the only internal remedy to which can be attributed a certain effect in psoriasis. Robinson[m] says it acts better in psoriasis when given with alkalies. Crocker[b] says arsenic should not be given in psoriasis, nor other skin diseases in which it is indicated, until all derangements of the general health have been rectified. Simon[f] says psoriasis may be cured simply by the administration of arsenic internally and the external use of soap and water, but relapses will occur no matter what treatment is employed. As most cases of this disease are essentially chronic and rarely come under observation until the acute stage is passed, arsenic may be commenced at once, and this comes near being the *one* disease in which we may always safely prescribe it, though pemphigus is "a good second."

PEMPHIGUS.—To Hutchinson, of London, is given the credit of having first directed attention to the beneficial effects of arsenic in this disease, but effect is now considered dependent both upon the stage and the variety of the disease. Roberts[j] says it "acts like a charm in some cases, but not in all." Hebra[c] (in 1884) said one could expect no benefit from internal remedies in pemphigus. Robinson[m] thinks it less beneficial in pemphigus foliaceus than in p. vulg. Bulkley[n] considers it almost a specific in this disease. Duhring[g] says arsenic is of great benefit in pemphigus, but we must distinguish between pemphigus and *pemphigoid* eruptions. Crocker[b] attributes action in this disease to its "vaso-motor" effect.

The disease which Duhring has described as *dermatitis herpetiformis*, and which is now generally recognized, though there has been a great deal of discussion as to the propriety of the name, is one in which there has seemed to be good effect from arsenic. Morrow[d] quotes a case of Robinson cured by arsenic. Piffard[o] thinks it the only drug which exercises the slightest influence in *relapsing herpes of the genitals.* Mentions, also, two cases of vesiculo-bullous syphilis, which made no improvement under mercury and iodide of potassium until arsenic was combined with them. He concludes that it exerts a very definite and specific influence in connection with disseminated vesicular and bullous *lesions*, irrespective of the cause.

LICHEN RUBER.—The elder Hebra[c] first introduced arsenic as a remedy in this disease, and Piffard[k] says the former's one case which recovered was under large doses of this drug. Duhring[g] says it is prescribed with great advantage in the later stages.

LICHEN PLANUS.—The German view is that the two diseases last mentioned are manifestations of the same process, while the American dermatologists, as a rule, consider them separate diseases, with Elliot, of New York, as a notable exception. Those who believe in the *identity* of the diseases will treat "lichen ruber planus," as they treat "lichen ruber acuminatus," internally with arsenic, though perhaps with the caution that it is best prescribed after acute stage is past. Robinson, who has written a long argument against their identity,[p] says [m] that arsenic should not be given in *lichen planus*, as it frequently aggravates the eruption. The *pityriasis rubra pilaris*, of Devergie,[q] has been said by continental authorities to be the disease which has been described by American writers as *lichen ruber*. Boeck, of Christiana, reported good results from arsenic in Devergie's disease,[r] although the French had found it of little value. However, whether the reported cases were lichen ruber or pityriasis rubra pilaris, arsenic has some reputation as a remedy.

We can close the list of skin diseases in which arsenic is generally admitted to be useful internally with the few already mentioned. I do not now remember any other in which a special cutaneous effect is at all unanimously conceded. As regards the great number remaining, opinions differ greatly concerning the value of arsenic as a remedy, hence we must conclude that it is prescribed in these cases either for its "tonic" effect, or else its use is purely empirical. A clear knowledge of its physiological effects should prevent our incurring the risk of being called empirical when we do prescribe it. It seems to me one has no more justification in calling arsenic an empirical remedy for psoriasis[k] than in saying the same of mercury in syphilis, or quinine in malarial disease. A review of the older and some of the later literature acquaints us with some more or less curious views concerning the action of arsenic. Neligan[l] says it is indicated either for obstinacy of the affection or where other remedies fail. Dr.

Hunt, who wrote twenty-five years ago, considered *naevus araneus* (a spider-shaped new growth of vessels) a constitutional disease, and treated it "successfully" through the administration of arsenic. Dr. Van Arcken[s] described a disease which he called *curate*, which was characterized by the occurrence of variously colored maculæ upon the skin, and he reported arsenic as one of the beneficial remedies. Buchner[t] has written an article in which he advanced the view that arsenic internally would act as a prophylactic against scarlatina. Hans Hebra[c] very well says the future will have to decide this question!

Dr. Bulkley, of New York, is probably the most liberal prescriber of arsenic in skin diseases, Fowler's solution being an ingredient of the majority of his tonic prescriptions. Unna[u] seems to admit some virtue in arsenic as a remedy for his seborrhoic eczema, but he qualifies the view by saying it does not act so well as in psoriasis, nor will it prevent recurrence without the aid of local prophylaxis. This disease, as a rule, is not characterized by as acute symptoms as the ordinary eczema.

HYPODERMICALLY.—Köbner is quoted[b] as having obtained good results from the employment of arsenic in this manner in multiple sarcomata. Funk[v] refers to this, recommends arseniate of sodium thus employed in idiopathic sarcoma cutis, and mentions numerous cases of pseudo-lenkæmia, multiple wart formation (Lewin), and "melanotic tumors" (Bonlay) as having been cured by this method of administration.

As conclusions drawn from the large number of authorities, I may add the following remarks: Arsenic is useful, or even "almost specific," in a few diseases of the skin, or certain stages of disease of the skin, but the prescribing of the remedy for every skin disease without regard for a diagnosis, and without consideration of the degree of irritation present is inexcusable empiricism. Even in cases where it might be indicated the condition of the alimentary tract will often preclude its use, as digestive disorder might be increased and, in many cases, produce bad consequences reflexly upon the skin. Many indolent or subacute diseases of the skin may be benefited, but often this alone will be an insufficient indication, and time and drugs will be wasted in fruitless groping in the dark. Arsenic must as surely be accorded as definite a place in definite diseases of the skin as is given to any remedy in our pharmacopœia, in any disease met by the general practitioner. The use of the drug empirically in indiscriminate skin affections must come to share the odium which attaches to "doctoring the blood," after the charlatan and patent medicine plan.

### BIBLIOGRAPHY.

*a*Bartholow. "Materia Medica and Therapeutics." 1884.

*b*Crocker. "Diseases of the Skin." 1888.

H. von Hebra. "Die Krankhaften Veränderungen der Haut und Ihrer Anhangsgebilde," etc. 1884.

*d*Morrow. "Atlas of Venereal and Skin Diseases." 1888–'89.

*e*Journal of Cutaneous and Venereal Diseases. Vol. VI. 1888.

*f*Simon. "Wood's Medical and Surgical Monographs." Vol. I. No. I. 1889.

*g*Duhring. "Diseases of the Skin." Third Ed. 1888.

*h*Stelwagon. "Essentials of Diseases of the skin." Saunders' Question Compends. 1890.

*i*"Neligan on Diseases of the Skin," 1852. Revised by Belcher, 1865.

*j*Roberts. "Practice of Medicine." Fifth Ed. 1884.

*k*Piffard. "Cutaneous and Venereal Memoranda." 1887.

*l*Van Harlingen. "Ann. of the Universal Medical Sciences." 1888.

*m*Robinson. "Manual of Dermatology." 1885.

*n*Bulkley. "Manual of Diseases of the Skin." 1887.

*o*Piffard. "Journ. of Cut. and Genito-Urinary Diseases." April, 1890.

*p*Robinson. "Journal. Cut. and Gen. Urin Dis." Jan., Feby. and March, 1889.

*q*Alph. Devergie. "Traité Pratique des Maladies de la Peau." 3d Ed. Paris, 1863, (Ref.)

*r*Cæsar Boeck. "Monatshefte für Praktische Dermatologie." Band VIII. No. 3.

*s*Van Arcken. "Am. Med. Monthly Journal." April, 1858.

*t*Buchner. "Eine neue Theorie über Erzielung von Immunität gegen Infectionskrankheiten," etc. 1883.

*u*Unna. "Journ. Gen. Ur. and Cut. Dis." Dec., 1887.

*v*Funk. Monatshefte für Prak. Derm. Vol. VIII. No. II.

